# Evaluation of Simplified Diet Scores Related to *C*-Reactive Protein in Heavy Smokers Undergoing Lung Cancer Screening

**DOI:** 10.3390/nu14204312

**Published:** 2022-10-15

**Authors:** Federica Sabia, Alessandra Borgo, Alessandra Lugo, Paola Suatoni, Daniele Morelli, Silvano Gallus, Anna Villarini, Ugo Pastorino

**Affiliations:** 1 Thoracic Surgery Unit, Fondazione IRCCS Istituto Nazionale Tumori, Via Venezian 1, 20133 Milan, Italy; 2 Department of Environmental Health Sciences, Istituto di Ricerche Farmacologiche Mario Negri IRCCS, 20156 Milan, Italy; 3 Laboratory Medicine Unit, Fondazione IRCCS Istituto Nazionale Tumori, 20133 Milan, Italy; 4 Department of Research, S.S.D. Valitative Epidemiology, Fondazione IRCCS Istituto Nazionale Tumori, 20133 Milan, Italy

**Keywords:** diet score, *C*-reactive protein, inflammation, heavy smokers, lung cancer screening

## Abstract

The aim of this study was to assess the relationship between adherence to a healthy diet, such as the Mediterranean diet (MedDiet), and *C*-reactive protein (CRP) in Italian heavy smokers undergoing an LDCT screening program (bioMILD trial), using scores calculated by simple questionnaires. Simple formats of food frequency questionnaires were administered to a sample of 2438 volunteers, and the adherence to a healthy diet was measured by the validated 14-point MEDAS and by two adaptations proposed by us: 17-item revised-MEDAS and 18-item revised-MEDAS. The OR of CRP ≥ 2 mg/L for 1-point increase in 14-point MEDAS score was 0.95 (95% CI 0.91–0.99), for 17-point score was 0.94 (95% CI 0.91–0.98), and for 18-point score was 0.92 (95% CI 0.88–0.97). These inverse associations remained statistically significant also after further adjustment for body mass index. These results showed the efficacy of simplified scores and their relationship with lower levels of CRP in a population of heavy smokers. This suggests that a targeted nutritional intervention might achieve a substantial reduction in CRP levels. The findings will be prospectively tested in a new randomized study on primary prevention during lung cancer screening.

## 1. Introduction

Lifestyle plays a central role in human health: tobacco smoking, inadequate diet, and sedentary behavior are the leading reversible causes of incidence and mortality for major chronic diseases [1,2,3]. High intake of red meat, processed foods, sugars, and sedentary lifestyles are associated with an increased risk of cancer, cardiovascular, and metabolic diseases. Instead, higher physical activity and a healthy diet have a protective effect, including high-risk populations, such as current smokers [1,4,5,6].

There is evidence that the adherence to the traditional Mediterranean diet (MedDiet), rich in plant-based foods (extra virgin olive oil, vegetables, fruits, whole grain cereals, nuts, and legumes) with moderate amounts of fish, poultry, eggs, and dairy products and low amounts of red meat and sweets, is inversely associated with higher body mass index (BMI) or weight gain [7,8,9] and has benefits in reducing several chronic diseases, such as cancers [10,11,12,13,14]. In addition, adherence to MedDiet could reduce systemic inflammation [15,16]. Several studies on the Mediterranean diet report a reduction of *C*-reactive protein (CRP) levels [17,18,19,20], the most common inflammatory marker used in routine clinical practice [21]. Elevated levels of circulating CRP are associated with an increased risk of lung cancer (LC) in cancer-free individuals, as well as low-dose computed tomography screening participants, and the value of CRP in predicting disease progression and response to therapy has been widely explored in the setting of chronic inflammatory, cardiovascular, and chronic obstructive pulmonary disease [22,23,24,25,26,27]. In particular, a direct association was found between elevated levels of CRP, and other inflammatory markers, with smoking intensity and/or duration [28,29,30].

Dietary scores can be useful tools for evaluating adherence to MedDiet [31,32]. The most used score was developed by Trichopoulou et al., which applies to semiquantitative food-frequency questionnaire (FFQ) including approximately 150 foods and beverages [33]. This tool is useful to determine the intake of individual foods, food groups, or nutrients, but it could be complex and time-consuming, and unsuitable for experimental or observational studies. As a consequence, several short screeners have been validated for a rapid assessment of MedDiet adherence [32,34,35]. To our knowledge, in Italy, only two questionnaires have been developed to measure compliance with the MedDiet: a 9-item questionnaire [36] whose validation has been conducted by comparing it with another score of only eleven items developed in Greece [37], and a more recent 15-item questionnaire validated against the European Prospective Investigation into Cancer and Nutrition Food Frequencies Questionnaire (EPIC-FFQ) [38]. However, none of these studies has so far related the dietary score to CRP levels.

In Spain, the PREvencion con DIeta MEDiterranea (PREDIMED) study developed and validated a 14-item Mediterranean Diet Adherence Screener (MEDAS) to assess dietary compliance quickly [32]. The relationship between MEDAS and CRP levels has been previously investigated in two studies on the Spanish population, one giving negative results [39] and one showing an inverse relationship between MedDiet adherence and CRP [40].

In the last two decades, several screening trials conducted on heavy smokers populations evaluated the effect of Low-Dose Computed Tomography (LDCT) screening on reducing overall and LC mortality [41,42,43,44,45,46,47,48]. Theoretically, prevention of LC incidence and mortality could be also achieved by smoking cessation as well as improving dietary habits.

The aim of this study was to test new formats of dietary questionnaires on Italian heavy smokers undergoing an LDCT screening program and to evaluate the relationship between Mediterranean dietary habits, measured by the MEDAS score and an adapted MEDAS, which includes food items commonly used in the Italian diet, and CRP levels. This study has been instrumental to define an anti-inflammatory nutritional intervention as primary prevention for heavy smokers volunteers enrolled in a new prospective LDCT screening program, the SMILE study (Screening and Multiple Intervention on Lung Epidemics, clinicaltrials.gov ID: NCT03654105).

## 2. Materials and Methods

### 2.1. Study Population

The present study population was extracted from subjects who participated in the bioMILD trial (clinicaltrials.gov ID:NCT02247453), an ongoing large prospective study of the Fondazione IRCCS Istituto Nazionale dei Tumori of Milan, that enrolled a total of 4119 heavy smoking volunteers (i.e., (i) current or ex-smokers from less than 10 years, with ≥30 packs/years, aged 50–75 years and free from neoplasms within the previous five years, or (ii) current or ex-smokers from less than 10 years, with <30 packs/years, aged 50–75 years and free from neoplasms within the previous five years with additional risk factors such as family history of lung cancer, prior diagnosis of chronic obstructive pulmonary disease (COPD) or pneumonia, professional exposure to known carcinogens) from January 2013 to March 2016 to undergo LDCT examination, spirometry, plasma and blood sample collection, and microRNA profiling.

More specifically, the present study is a secondary analysis of the main bioMILD trial. Out of 4119, we selected 3810 (93%) alive participants on May 2018 with an available e-mail address to collect information on dietary habits.

The study was approved by the Ethics Committee of the Istituto Nazionale Tumori of Milan (code: INT 0021/11) and performed in accordance with relevant guidelines and regulations. All the eligible patients signed an informed consent form before enrollment.

### 2.2. Subjects Data Collection

Available data from baseline bioMILD questionnaires and examinations included age, gender, smoking habits, educational level, BMI, information on chronic diseases and drug consumption, and forced expiratory volume in the first second of expiration (FEV1). All participants underwent pre-bronchodilator spirometry using a flow spirometer (KoKo; nSpire Health, Longmont, CO, USA) according to ATS/ERS criteria.

### 2.3. Blood Sample Collection and CRP Mark

Plasma was collected at baseline for each participant and CRP level was quantified by immunoturbidimetry using a Roche automated clinical chemistry analyzer (Cobas C 6000, Roche Diagnostics, Belleville, NJ, USA).

### 2.4. Lifestyle Questionnaires

The selected participants were invited by e-mail to fill out newly administered questionnaires including specific items on dietary habits. Four different types of questionnaires were sent to an equal number of subjects, randomly selected from the study population with similar epidemiologic characteristics. The four formats of the questionnaires were: (a) long radio buttons question type (select one single answer option in a list of alternatives) with 40 questions, (b) short radio buttons question type with 20 questions, (c) long free text question type with 40 questions, and (d) short free text question type with 20 questions.

In particular, subjects were asked to indicate the average daily/weekly consumption or types of selected foods and food groups.

For the present analysis, we evaluated all information retrieved from the four types of questionnaires.

### 2.5. MedDiet Screener

The adherence to the MedDiet was measured by the 14-item Mediterranean Diet Adherence Screener (MEDAS), developed by PREDIMED trial [32]. The MEDAS consists of 14 questions on food intake habits, and each question is scored 0 or 1 to calculate a final MEDAS score ranging from 0 to 14 (Table S1). The MEDAS score was revised by adding up to 4 items available from the lifestyle questionnaires administered to the study population and not already included in the MEDAS score, related to foods that showed the major pro- or anti-inflammatory potential (Table S2). Questions on sugar, whole grain cereals, and orange vegetables/fruits are in all four different formats of the questionnaire and each item was scored 0 or 1. Each point was added to the 14-item MEDAS to determine a 17-item revised-MEDAS. This score was calculated on the whole study population (2438 participants). The question on coffee consumption was only present in the two long-format questionnaires, and the assigned score 0 or 1 was combined with the previous 17-item revised-MEDAS to calculate an 18-item revised-MEDAS, available only for subjects with the long-format questionnaire (1219).

### 2.6. Statistical Analysis

Outlier values of self-reported dietary information of the free text question type questionnaires were handled by a multiple imputation analysis using the fully conditional specification model for arbitrary missing patterns that assumes the existence of a joint distribution for all variables [49]. As a rule of thumb, the number of imputed datasets should be at least equal to the percentage of incomplete cases [50]. The questionnaires were compared on the basis of response and completion rate. The differences between strata were tested by Chi-squared tests with a significance level equal to 0.05.

The CRP level was dichotomized into low (<2 mg/L) and high (≥2 mg/L) according to previous experience [22,23,24,25]. Regression coefficients and odds ratios (OR), with corresponding 95% confidence intervals (CI) and *p*-values, for high versus low CRP levels were estimated using multivariate unconditional logistic regression models to evaluate the relationship between CRP levels and the original 14-item MEDAS score and the two adapted versions of MEDAS score with the related added items after adjusting for sex, age (continuous), pack-years (categorical, <30 and ≥30), smoking status, FEV_1_% (categorical, ≤90 and >90 [25]), chronic diseases, taking metformin (yes/no), taking a statin (yes/no), taking Acetylsalicylic acid (ASA) (yes/no), BMI, and type of questionnaire completed. BMI was categorized into underweight, normal weight, overweight, and obesity according to the standard WHO categorization.

Multivariate unconditional logistic regression models for higher CRP levels according to BMI categories, after adjustment for the same covariates as above except for the type of questionnaire completed, and results were reported in Supplementary Materials.

Statistical analyses were carried out using Statistical Analysis System Software (version 9.4; SAS Institute, Cary, NC, USA).

## 3. Results

### 3.1. Study Sample

Of the 3810 questionnaires sent to bioMILD trial participants (i.e., 964 long radio buttons question type, 943 short radio buttons question type, 952 long free text question type, and 951 short free text question type), 2635 (69.2%) were returned and 2438 were complete (92.5% of returned and 64.0% of eligible). No difference was observed among the four types of questionnaires in terms of response rate (68.4% of long radio buttons question type, 68.5% of short radio buttons question type, 70.2% of long free text question type, and 69.6% of short free text question type, *p* = 0.796) and completion (93.2%, 93.5%, 90.6%, and 92.9% respectively, *p* = 0.164). Outlier values of reported food’s intakes of the free text question types were 1% of all answers.

The distribution of selected characteristics of the 2438 volunteers at baseline is described overall and stratified by questionnaire randomly assigned (Table 1). The median age of the study participants was 59 (IQR: 54–64); they were mainly men and current smokers, with median pack-years of 41 (IQR: 34–51). The 56% had an intermediate educational level (secondary school and vocational course). The median CRP level was 1.3 mg/L (IQR: 0.63–2.59), the median BMI was 25.4 kg/m^2^ (IQR: 22.86–27.92), and 35% of the selected population had a prior diagnosis of chronic diseases.

### 3.2. MedDiet Scores and CRP

The original 14-item MEDAS score showed a statistically significant protective association with higher CRP level (Table 2). Adjusted regression coefficient β and OR for a 1-point score increment were respectively −0.05 and 0.95 (95%CI: 0.91–0.99, *p*= 0.0282). Results of adjusted logistic regression of revised-MEDAS scores and their added items are shown in Table 2. The consumption of <2 tsp per day of sugar was inversely related to high CRP (β = −0.17, OR 0.85, 95%CI 0.71–1.01, *p* = 0.0635). Subjects who consumed whole grain cereals were more likely to have lower CRP levels (β = −0.18, OR 0.84, 95%CI 0.71–1.00, *p* = 0.0549). More than 1 serving per week of orange vegetables and fruits was inversely related to higher CRP level (β = −0.20, OR 0.82, 95%CI 0.67–1.00, *p* = 0.0559). The three estimates are not statistically significant but on the limit of significance. 1-point increment of the 17-item revised-MEDAS score showed a statistically significant inverse association with higher CRP levels, with regression coefficient β and OR respectively −0.06 and 0.94 (95%CI: 0.91–0.98, *p*= 0.0033). Analysis only on long-format questionnaires showed that 2 or more servings per day of coffee had a protective effect on CRP levels (β = −0.48, OR 0.62, 95%CI 0.43–0.89, *p* = 0.0091). The 18-item revised-MEDAS with its 1-point increment showed a statistically significant inverse association with CRP levels, β −0.08 and OR 0.92 (95%CI 0.87–0.97, *p* = 0.0022).

Multivariate regression according to BMI levels showed that as BMI increased, the prevalence of high CRP levels was significantly higher (Table S3).

## 4. Discussion

In our population of Italian heavy smokers enrolled in an LDCT screening trial (bioMILD), the adherence to MedDiet, measured by a score, was inversely related to CRP levels.

The effect of Mediterranean eating habits on CRP concentrations has been investigated in several studies. Most of them used long and complex FFQ (over 80 questions). We tested shorter formats of questionnaires as a simple and quick tool to guarantee the most possible compliance of our selected population, who completed the questionnaires online independently. Compared to the validated MEDAS, a simple questionnaire of 14 items, we increased the number of questions by separating more items and investigating more food consumptions to overcome the declared limits of too few questions and to be able to improve it with further items [40]. Our questionnaires investigated the consumption of typical Mediterranean foods and other foods whose consumption is frequent in the Italian population (e.g., coffee) and/or which the literature indicates an anti-inflammatory or pro-inflammatory potential. We administered four different questionnaires, 20 or 40 questions and radio button or free text questions, to test which one guarantees greater compliance by the population and to test the most useful one to determine a more reliable score to investigate the food habits of the Italian population, which represents our target population.

The predictive utility of MEDAS score was validated not only in the Spanish population but also in other countries (e.g., UK, USA, Germany) without modifications [51,52,53]. A local adaptation of MEDAS was made in the Israeli population, where a new I-MEDAS score was calculated from FFQ data to reflect national dietary recommendations in Israel [54].

We not only applied the original 14-item MEDAS score, but we also improved it by including selected food items commonly used in the Italian diet that, according to literature, have shown a potential pro-inflammatory or anti-inflammatory effect in multivariate regressions for high CRP versus low CRP, adjusted for BMI, which is directly related to CRP levels as our analysis in Supplementary Materials confirmed, and other covariates. We created a 17-item revised MEDAS by adding questions on sugar, whole grain cereals and orange vegetables and fruits. The Mediterranean diet is characterized by a high intake of complex carbohydrates in the form of whole grains, characterized by a low glycemic index where the sugar comes from the fruit alone. Whole grain cereals showed an anti-inflammatory effect [55]. Instead, an excess of sugar has been linked to inflammation [56]. The consumption of orange fruit and vegetables rich in carotenoids has been evaluated for their potential protective effect against lung cancer [1]. An 18-item revised MEDAS score was created by adding a further question about coffee consumption, widely consumed by Italians, which showed to be protective against high CRP [57]. We observed an inverse association between the original 14-item MEDAS score and CRP, as previously shown in the Spanish population [40]. The statistically significant inverse association with CRP was also observed with a slightly greater significance for the two adapted scores, 17-item revised-MEDAS and 18-item revised-MEDAS, which investigated more dietary components. In the 18-item revised-MEDAS, calculated only on half of the population to which long-format questionnaires were assigned, the association with CRP retained the same significance as the 17-item revised-MEDAS, despite the smaller number of subjects.

Our findings suggested that an adapted MedDiet score, adding a few inflammation-related foods, was more effective to investigate the relationship between diet and CRP in our Italian heavy-smoking population.

Furthermore, there was no difference in the response rate between the four types of questionnaires used. A long free text question type with 40 questions appeared to be the preferable option, to refine the score with more questions and modified cut-offs of serving size while remaining a simple format.

The main limitations of our study are those inherent to the cross-sectional design, including in particular the inability to derive causal inference. Strengths of the study include the large sample size and the availability of objective markers (i.e., CRP), which were determined in the same laboratory for all subjects.

Such evaluation has been instrumental to the design of a prospective randomized trial combining lung cancer screening with primary prevention: the SMILE trial. The SMILE study is an ongoing trial launched in 2019 at the Istituto Nazionale dei Tumori of Milan, and it tests the feasibility and efficacy of multiple interventions, including targeted dietary modification and physical activity, in addition to smoking cessation with cytisine treatment, reduction of chronic inflammation by low-dose aspirin (ASA), and early diagnosis with LDCT. Each randomized heavy smoker volunteer was assigned a personal account on the SMILE website (https://www.programmasmile.it, accessed on 10 August 2022), and every six months, all participants were invited to complete online free text format questionnaires on eating and lifestyle habits. The SMILE trial was approved by the Ethics Committee of the Istituto Nazionale Tumori of Milan and the Italian Medicines Agency (AIFA). Positive results on smoking cessation therapy with cytisine at one year of follow-up have already been published [58]. Analysis of the efficacy of the nutritional intervention will be published at the end of 2 years of follow-up.

## 5. Conclusions

Our data showed a close inverse relationship between adherence to MedDiet and lower levels of CRP in a population of heavy smokers, including the efficacy of a simplified dietary score. These results suggest that targeted interventions on dietary habits could modify CRP levels, and reduce the overall mortality risk of heavy smokers. An anti-inflammatory nutritional intervention could be offered as a standard activity in future LDCT screening programs to strengthen screening efficacy.

## Figures and Tables

**Table 1:** Baseline selected characteristics of 2438 participants according to the questionnaire’s type.

		All Subjects	Free Text Question Type		Radio Buttons Type	
			Long	Short	Long	Short
		*n*	(%)	(%)	(%)	(%)
**Total**		2438	24.8	25.2	25.2	24.8
**Sex**	**Male**	1534	62.5	61.1	61.4	66.7
	**Female**	904	37.5	38.9	38.6	33.3
**Age**	**<55 years**	624	21.8	27.2	27.2	26.2
	**55–64 years**	1307	58.0	51.7	51.5	53.3
	**≥65 years**	507	20.2	21.1	21.3	20.5
	**Median (IQR)**	59 (54–64)	59 (55–64)	58 (54–64)	59 (54–64)	59 (54–63)
**Pack-Years**	**<30**	177	7.8	5.7	8.0	7.6
	**≥30**	2261	92.2	94.3	92.0	92.4
	**Median (IQR)**	41 (34–51)	42 (35–52)	41 (35–50)	42 (34–52)	40 (34–50.5)
**Smoking status**	**Former**	587	25.1	25.8	21.8	23.5
	**Current**	1851	74.9	74.2	78.2	76.5
**Fev1%**	**>90%**	1600	62.2	66.0	67.1	67.2
	**≤90%**	838	37.9	34.0	32.9	32.8
	**Median (IQR)**	96.00 (86–106)	95 (86–105)	97 (87–107)	96 (86–105)	97 (87.5–106)
**CRP**	**<2 mg/L**	1605	65.0	69.8	64.8	63.7
	**≤25 mg/L**	581	23.0	22.3	24.9	25.2
	**>5 mg/L**	252	12.1	8.0	10.3	11.1
	**Median (IQR)**	1.34	1.36	1.21	1.39	1.40
		(0.63–2.59)	(0.66–2.59)	(0.57–2.47)	(0.65–2.64)	(0.65–2.74)
**BMI**	**Underweight**	38	2.0	1.5	1.6	1.2
	**Normal weight**	1065	42.0	45.2	43.5	44.0
	**Overweight**	1022	41.5	42.1	42.0	42.1
	**Obese**	313	14.6	11.2	12.9	12.8
	**Median (IQR)**	25.39	25.51	25.26	25.39	25.40
		(22.86–27.96)	(22.98–28.06)	(22.99–27.78)	(22.83–28.08)	(22.94–27.73)
**Chronic Diseases ^a^**		859	36.7	34.2	36.8	33.3
**Medications used**						
	**ASA**	382	17.0	13.5	16.0	16.2
	**Metformin**	157	7.1	6.2	6.5	6.0
	**Statins**	359	14.9	15.1	14.3	14.6

**IQR**, Interquartile Range. **CRP**, *C*-Reactive protein. **FEV_1_%**, Forced Expiratory Volume in one second percent. **BMI**, Body Mass Index, Underweight (<18.5), Normal weight (18.5–24.9), Overweight (25–29.9), Obese (≥30). **ASA**, Acetylsalicylic acid. ^a^ Chronic disease: Lung diseases (COPD, emphysema, chronic bronchitis, asthma, active sarcoidosis), Cardiovascular diseases (heart attack, angina, heart failure, stroke, atherosclerosis, TIA, diabetes, atrial fibrillation, aortic aneurysm, peripheral vasculopathy), Autoimmune diseases (Basedow, thyroiditis, chronic hepatitis, nephritis/glomerulus, nephritis, Crohn’s disease, ulcerative rectocolitis, severe obesity, vasculitis, rheumatoid arthritis), Infectious Diseases (HCV, HIV, chronic hepatitis (not A)), other chronic diseases (renal failure, polycythemia, myelofibrosis, essential thrombocythemia, hematological diseases), advanced Active Tumor.

**Table 2:** Multivariable logistic regression model of *C*-Reactive Protein (CRP) ≥ 2 mg/L versus CRP < 2 mg/L according to: (a) the original 14-item MEDAS score, (b) 17-item revised-MEDAS score and its single added food items, and (c) 18-item revised-MEDAS and the added food item.

	N. Subjects	CRP ≥ 2 mg/L vs. CRP < 2 mg/L ^a^		
			(34.2%)	
		Regression Coefficient β	OR (95%CI)	*p*
**14-item MEDAS score (1-point increase)**	2438	−0.05	0.95 (0.91–0.99)	0.0282
Sugar < 2 vs. ≥2 tps/day	2438	−0.17	0.85 (0.71–1.01)	0.0635
Whole grain cereals ≥1 vs. <1 serving/week	2438	−0.18	0.84 (0.71–1.00)	0.0549
Orange vegetables and fruits ≥2 vs. <2 serving/week	2438	−0.20	0.82 (0.67–1.00)	0.0559
**17-item revised-MEDAS (1-point increase)**	2438	−0.06	0.94 (0.91–0.98)	0.0033
Coffee ≥2 vs. <2 serving/day ^b^	1219	−0.48	0.62 (0.43–0.89)	0.0091
**18-item revised-MEDAS ^b^ (1-point increase)**	1219	−0.08	0.92 (0.88–0.97)	0.0022

**CRP**, *C*-Reactive protein. **OR**, Odds Ratio. **CI**, confidence intervals. ***p***, *p*-values. ^a^ Regression coefficients and ORs were estimated using unconditional multiple logistic regression models after adjustment for sex, age, pack-years, smoking status, FEV, chronic diseases, metformin, statin, Acetylsalicylic acid, BMI, and type of questionnaire. ^b^ Data available only from long questionnaires (*n* = 1219).

## Data Availability

Not applicable.

## Supplementary Materials

The following supporting information can be downloaded at: https://www.mdpi.com/article/10.3390/nu14204312/s1, Table S1: The original Mediterranean Diet Adherence Screener (MEDAS) items and score criteria. Table S2: Selected food items and score criteria added to the original MEDAS for the revised-MEDAS scores. Table S3: Odds ratio (OR) of *C*-Reactive Protein (CRP) ≥ 2 mg/L versus CRP < 2 mg/L, and corresponding 95% confidence intervals (CI), according to body mass index (BMI) value.
