# Supplementary material for: Evaluation of Simplified Diet Scores Related to C-Reactive Protein in Heavy Smokers Undergoing Lung Cancer Screening
Source: Nutrients. 2022 Oct 15;14(20):4312. doi: 10.3390/nu14204312 (PMC9610125; doi:10.3390/nu14204312)
Supplement: Supplementary file 1 [file nutrients-14-04312-s001.zip › nutrients-1935344-supplementary.pdf]

Supplementary Materials

Evaluation of Simplified Diet Scores Related to C-Reactive Protein in Heavy Smokers Undergoing Lung Cancer Screening

Table S1. The original Mediterranean Diet Adherence Screener (MEDAS) items and score criteria.

**Question**	**Criterion to score 1 point**
1. Do you use olive oil as the principal source of fat for cooking?	Yes
2. How much olive oil do you consume per day (including that used in frying, salads, meals eaten away from home, etc.)?	≥4 tbsp
3. How many servings (80 g for salads and 200 g for others) of vegetables do you consume per day?	≥2
4. How many pieces of fruit (including fresh-squeezed juice) do you consume per day?	≥3
5. How many servings (100 g) of red meat, hamburger, or sausages do you consume per day?	<1
6. How many servings (12 g) of butter, margarine, or cream do you consume per day?	<1
7. How many carbonated and/or sugar-sweetened beverages do you consume per day?	<1 glass
8. Do you drink wine? How much do you consume per week?	≥7 cup (125 ml)
9. How many servings (150 g) of legumes do you consume per week?	≥3
10. How many servings (150 g of fish and 200g of seafood) of fish/seafood do you consume per week?	≥3
11. How many times do you consume commercial (not homemade) pastry such as cookies or cake per week?	<3
12. How many times do you consume nuts per week? (30 g)	≥3
13. Do you prefer to eat chicken, turkey or rabbit instead of beef, pork, hamburgers, or sausages?	Yes
14. How many times per week do you consume boiled vegetables, pasta, rice, or other dishes with a sauce of tomato, garlic, onion, or leeks sautéed in olive oil?	≥2

tbsp, tablespoon.

Table S2. Selected food items and score criteria added to the original MEDAS for the revised-MEDAS scores.

Question	Criterion to score 1 point
15. How much sugar do you consume per day?	<2 tsp
16. How many servings (80 g pasta/rice and 50 g bread) of whole grain cereals do you consume per week?	≥1
17. How many servings (200 g vegetables and 150 g fruits) of orange vegetables and fruits do you consume per week?	≥2
18. How much coffee do you consume per day? [a]	≥2 cup

tsp, teaspoon.

[a] Data available only from long questionnaires (n=1219).

Table S3. Odds ratio (OR) of C-Reactive Protein (CRP) ≥2 mg/L versus CRP <2 mg/L, and corresponding 95% confidence intervals (CI), according to body mass index (BMI) value.

		All subjects<br>N	CRP ≥2 mg/L<br>%	OR (95%CI) [a]<br>CRP ≥2 mg/L vs. CRP <2 mg/L
**Total**		**2438**	**34.2**	
**BMI**				
	**Underweight**	38	18.4	0.69 (0.30-1.60)
	**normal weight**	1065	24.4	Ref
	**Overweight**	1022	37.9	**1.90 (1.55-2.32)**
	**Obese**	313	57.2	**4.27 (3.24-5.64)**

BMI, Body Mass Index. **CRP**, C-Reactive protein. **OR**, Odds Ratio**. CI,** confidence intervals.

[a] ORs were estimated using unconditional multiple logistic regression models after adjustment for sex, age, pack-years, smoking status, FEV_1%, chronic diseases, metformin, statin, and Acetylsalicylic acid.
